# Biofilms inactivate the free-living stage of *Batrachochytrium dendrobatidis*, the most destructive pathogen for vertebrate diversity

**DOI:** 10.1093/ismejo/wrae189

**Published:** 2024-09-26

**Authors:** Hugo Sentenac, Dirk S Schmeller, Solène Caubet, Adélaïde Carsin, Rémi Guillet, Jessica Ferriol, Joséphine Leflaive, Adeline Loyau

**Affiliations:** Centre de Recherche sur la Biodiversité et l’Environnement (Unité Mixte de Recherche UMR 5300), Université de Toulouse, Centre National de la Recherche Scientifique (CNRS), Institut de Recherche pour le Developpement (IRD), Institut National Polytechnique de Toulouse (INPT), Université Toulouse 3—Paul Sabatier, 118 Route de Narbonne, Toulouse Cedex 31062, France; Centre de Biologie pour la Gestion des Populations (CBGP), Université de Montpellier, Centre de coopération internationale en recherche agronomique pour le développement (CIRAD), Institut national de recherche pour l’agriculture, l’alimentation et l’environnement (INRAE), Institut Agro, IRD, Avenue du Campus Agropolis, Montferrier-sur-Lez 34980, France; Chrono-Environnement UMR 6249 CNRS/Université de Franche-Comté (UFC), 16 Route de Gray, Besançon 25000, France; Centre de Recherche sur la Biodiversité et l’Environnement (Unité Mixte de Recherche UMR 5300), Université de Toulouse, Centre National de la Recherche Scientifique (CNRS), Institut de Recherche pour le Developpement (IRD), Institut National Polytechnique de Toulouse (INPT), Université Toulouse 3—Paul Sabatier, 118 Route de Narbonne, Toulouse Cedex 31062, France; Centre de Recherche sur la Biodiversité et l’Environnement (Unité Mixte de Recherche UMR 5300), Université de Toulouse, Centre National de la Recherche Scientifique (CNRS), Institut de Recherche pour le Developpement (IRD), Institut National Polytechnique de Toulouse (INPT), Université Toulouse 3—Paul Sabatier, 118 Route de Narbonne, Toulouse Cedex 31062, France; Centre de Recherche sur la Biodiversité et l’Environnement (Unité Mixte de Recherche UMR 5300), Université de Toulouse, Centre National de la Recherche Scientifique (CNRS), Institut de Recherche pour le Developpement (IRD), Institut National Polytechnique de Toulouse (INPT), Université Toulouse 3—Paul Sabatier, 118 Route de Narbonne, Toulouse Cedex 31062, France; Centre de Recherche sur la Biodiversité et l’Environnement (Unité Mixte de Recherche UMR 5300), Université de Toulouse, Centre National de la Recherche Scientifique (CNRS), Institut de Recherche pour le Developpement (IRD), Institut National Polytechnique de Toulouse (INPT), Université Toulouse 3—Paul Sabatier, 118 Route de Narbonne, Toulouse Cedex 31062, France; Centre de Recherche sur la Biodiversité et l’Environnement (Unité Mixte de Recherche UMR 5300), Université de Toulouse, Centre National de la Recherche Scientifique (CNRS), Institut de Recherche pour le Developpement (IRD), Institut National Polytechnique de Toulouse (INPT), Université Toulouse 3—Paul Sabatier, 118 Route de Narbonne, Toulouse Cedex 31062, France; Centre de Recherche sur la Biodiversité et l’Environnement (Unité Mixte de Recherche UMR 5300), Université de Toulouse, Centre National de la Recherche Scientifique (CNRS), Institut de Recherche pour le Developpement (IRD), Institut National Polytechnique de Toulouse (INPT), Université Toulouse 3—Paul Sabatier, 118 Route de Narbonne, Toulouse Cedex 31062, France; Centre de Recherche sur la Biodiversité et l’Environnement (Unité Mixte de Recherche UMR 5300), Université de Toulouse, Centre National de la Recherche Scientifique (CNRS), Institut de Recherche pour le Developpement (IRD), Institut National Polytechnique de Toulouse (INPT), Université Toulouse 3—Paul Sabatier, 118 Route de Narbonne, Toulouse Cedex 31062, France

**Keywords:** amphibian chytridiomycosis, biotic environmental factors, disease ecology, eco-epidemiology, emerging infectious disease, invasibility, mountain freshwater ecosystems, parasite, resilience

## Abstract

Emerging infectious diseases threaten biodiversity and human health. Many emerging pathogens have aquatic life stages and all immersed substrates have biofilms on their surface, i.e. communities of microorganisms producing a gelatinous matrix. However, the outcome of the interactions between environmental biofilms and pathogens is poorly understood. Here, we demonstrate that biofilms reduce the survival of the most impactful pathogen for vertebrate diversity, the invasive chytrid fungus *Batrachochytrium dendrobatidis*. Effects on its zoospores varied with biofilm composition in controlled settings and biofilm compositional variation also coincided with divergent impacts of chytridiomycosis on amphibian populations in nature. Our results suggest that biofilms form a biotic component of ecosystem resistance to *Batrachochytrium dendrobatidis* by reducing environmental transmission, and that they could be used to develop nature-based technologies to limit the impacts and spread of this invasive chytrid fungus. Our study warrants further research into the interactions between environmental biofilms and pathogenic and/or invasive micro-organisms.

## Graphical Abstract

**Graphical Abstract:**
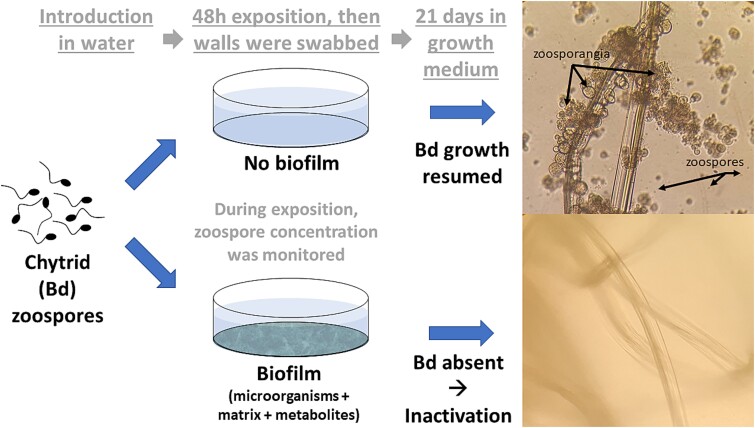

## Introduction

Emerging infectious diseases threaten human health, food security, and biodiversity [1, 2]. A good understanding of pathogen ecology is thus essential to elucidate the determinants of infection and diseases and provide effective mitigation strategies [3]. However, the impacts of biotic environmental factors on infectious agents have rarely been studied, whereas those agents that have a free-living stage must survive exposure to many sympatric organisms and the metabolites they produce to infect a new host [4, 5]. That is particularly true in aquatic environments, where sympatric organisms include a plethora of microorganisms, which often live in matrix-enclosed communities, or biofilms [6]. Biofilms are abundant and diverse in aquatic ecosystems but our knowledge of how infectious agents interact with them remains limited [7]. Some biofilms can shelter human infectious agents from predators and biocides, and serve as a reservoir for pathogenic bacteria, viruses, or protozoans [8, 9]. In contrast, biofilms can constitute a sink for infectious agents, where the latter can be entrapped in the polymeric matrix, and then consumed, outcompeted, or inactivated by other biofilm residents or their metabolites [10, 11]. By exploiting these capabilities, biotechnologies based on biofilms (e.g. biofilters) have been developed to eliminate waterborne pathogens from the aquatic environment [12, 13]. Biofilm effects extend outside the matrix, as biofilms capture nutrients and can produce compounds with antimicrobial or allelopathic effects [7].

Amphibian chytridiomycosis, an emerging infectious disease, is the most destructive vertebrate disease ever recorded [14]. It is caused by *Batrachochytrium dendrobatidis* (Bd) [15], an aquatic fungus which parasitizes the skin of metamorphosed amphibians and the mouthparts of larval stages [16]. The infective stage of Bd is a free-living flagellated zoospore (body: 3–5 μm, flagellum 19–20 μm) able to swim in aquatic environments to find a new host via chemotaxis, thus enabling environmental transmission [17, 18]. Yet, the ability of Bd zoospores to infect a new host is constrained by the distance they can swim (<2 cm in still water in 24 h) and the time before they die or attach (usually attachment occurs before 24 h) which is ultimately dependent on biotic and abiotic environmental conditions [19]. When conditions are not favorable (e.g. lack of nutrients or temperature >25°C), zoospores quickly attach on proximate surfaces to maximize their survival: they persist in a state of inactivity but still stay infective for a period (up to 7 weeks) that depends at least upon nutrient availability [20]. *In vivo* and *in vitro* studies of the amphibian skin microbiome revealed that zoospores can be inactivated by microorganisms producing antifungal compounds [21–23]. Finally, during their life outside the host, zoospores can be consumed by planktonic filter-feeders including ciliates, rotifers, tardigrades, and crustaceans (such as cladocerans, ostracods, and copepods); this phenomenon was shown to reduce infection pressure for amphibians and even drive infection prevalence in natural settings [24, 25]. A negative impact of high densities of planktonic green algae on Bd zoospore abundance was also reported [24], suggesting physical interference or allelopathy. Currently, there are no studies on the interactions between Bd zoospores and sessile environmental microbiomes such as biofilms.

Free-living zoospores of Bd are likely to be abundant at the bottom of the water column as tadpoles of many amphibian species extensively feed on benthic biofilms [26]. Benthic biofilms may reduce zoospore numbers in water by increasing the rate at which they attach and/or die, through mechanisms including physicochemical interference of the matrix (entrapment), allelopathy, nutrient depletion, or consumption by biofilm dwellers. Some filter-feeding eukaryotes like rotifers and ciliates can be sessile or semi-sessile in biofilms, where they feed on planktonic organisms, including zoosporic chytrid fungi [27, 28]. Therefore, biofilms could limit environmental transmission of Bd infection, and thus reduce infection pressure, with a subsequent decrease in prevalence as well as infection burdens, and act as a biological barrier [25]. Mountain freshwater ecosystems offer a unique opportunity to study the potential importance of biofilms in the epidemiology of Bd infections. In the French Pyrenees, chytridiomycosis has been well studied [25, 29] with evidence of diverging disease dynamics—epizootic or enzootic—even in geographically close sites and populations of the same susceptible species (*Alytes obstetricans*) [30]. Benthic biofilms are abundant in mountain freshwaters, as light can penetrate to the bottom in these generally clear lakes and ponds [7] and, at least in the French Pyrenees, their composition greatly varies between sites [31]. Therefore, biofilms may contribute to explaining the site-specific component of Bd infections dynamics observed in this and other mountain ranges [32].

We combined field and laboratory approaches to test whether biofilms interact with Bd and play a role in the epidemiology of chytridiomycosis. First, we investigated associations between Bd infection dynamics (enzootic vs. epizootic) and the diversity, stability, and composition of biofilms, leveraging field data from the Pyrenees. Then, we ran a series of five laboratory experiments to unravel the effects of benthic biofilms on the persistence of Bd zoospores in the water column. In the first experiment, we exposed Bd zoospores to natural biofilms (imported from the field) that contained filter-feeding micro-eukaryotic organisms able to consume Bd zoospores and determined zoospore disappearance rates. We repeated the experiment with a semi-natural biofilm grown in the laboratory that likely contained consumers. Then, we used simple artificial biofilms without Bd consumer, made of only one diatom (*Nitzschia palea* or *Mayamea permitis*) or one cyanobacterium (*Leptolyngbya* sp.), and also examined the impact of a biofilm made of a mixture of these three phototrophic organisms. We expected biofilms to have a negative effect on Bd zoospores mainly through consumption by biofilm filter-feeders.

## Materials and methods

### Field study

#### Data collection and preparation

We sampled biofilms (*n* = 46) between 2016 and 2020 by scraping immersed rocks (15–30 cm deep) with a metal spatula previously disinfected with chlorhexidine and rinsed with sterile water, in five geographically-clustered mountain lakes with *A. obstetricans* population continuously infected by Bd since at least 2004, but with different long-term populational impacts of chytridiomycosis. After severe declines coinciding with the emergence of Bd, *A. obstetricans* populations of three lakes (Lhurs, Acherito, and Puits) showed stable abundance levels in spite of infection, which is typical of an enzootic dynamic. In contrast, populations of Arlet and Ansabere are continually declining and the very few *A. obstetricans* tadpoles sampled in recent years in these two lakes exhibited high Bd burden, consistent with an epizootic infection dynamic [30]. Ansabere and Acherito (~1.75 km apart) and, to a lesser extent, Arlet and Puits (4.9 km with little altitudinal gradient) are close to each other but still exhibit different disease dynamics (epizootic vs. enzootic, respectively). Thus, genetic differences alone are unlikely to drive the differences in enzootic vs. epizootic dynamics. Two biofilm samples were collected in each lake in 2016 (early and late summer), three in 2017 and 2018, and one in 2019 (except Puits) and 2020 (in 2020 for Puits and Arlet only). Basic lake description and local parameters at the time of sampling are given in Table S1 and Fig. S1, which show that abiotic aquatic conditions are not the cause of the observed epidemiological trends either. Biofilm samples were frozen on dry ice directly in the field. Biofilm deoxyribonucleic acid was extracted and purified from 400 mg of thawed sample using the NucleoSpin Soil kit (Macherey-Nagel, Düren, Germany) according to the manufacturer protocol. We amplified the V3 and V4 regions of the 16S ribosomal ribonucleic acid (rRNA) gene (F: 5′-CCTACGGGNGGCWGCAG and R:5′-GACTACHVGGGTATCTAATCC [33]); and the V8 and V9 regions of the 18S rRNA gene (F: 5′-ATAACAGGTCTGTGATGCCCT and R: 5′-CCTTCYGCAGGTTCACCTAC [34]), respectively, by PCR (protocols given in Supplementary information).

Products were sequenced on a MiSeq system (Illumina; 2 × 250 bp V3). Demultiplexing and the removal of primer and adapter sequences were performed using Cutadapt v3.4 [35]. Additional trimming, formation of contiguous sequences, identification of unique amplicon sequence variants (ASVs), and chimera removal were performed in R v4.2.0 [36] using the DADA2 v1.20.0 pipeline [37]. Taxonomy of ASVs of both 16S and 18S rRNA genes was assigned using SINA v1.7.2 and the SILVA 138.1 reference database [38,39]. For the 16S rRNA gene library, ASVs unclassified at the class level, or (mis)classified as eukaryotes, chloroplasts, or mitochondria were removed in R with the phyloseq package [40]. For the 18S rRNA library, unclassified ASVs at the superkingdom or superphylum levels as well as metazoan taxa belonging to Vertebrata, Arthropoda, Platyhelminthes, Annelida, and Mollusca were removed. Prokaryotic and micro-eukaryotic libraries were also cleaned by removing rare ASVs (ASVs not having at least five reads in at least five samples were removed). We applied a centered-log-ratio (clr) transformation on this filtered dataset and used the Aitchison distance to measure pairwise dissimilarity [41, 42].

### Statistical analyses

Several biofilm attributes were compared between the two groups (epizootic vs. enzootic). First, we tested for differences in α-diversity using linear mixed models (LMM, with package lmerTest [43]), with the Chao1 index as response variable, the grouping variable as fixed effect, and lake as random effect. The Chao1 index (estimated richness) was determined before filtering out rare ASVs with vegan [44, 45]. Second, to investigate compositional differences between groups (β-diversity), we implemented a permutational multivariate analysis of variance (PERMANOVA), using the vegan function adonis2 and 999 permutations [46]. We checked for multivariate overdispersion with the vegan function betadisper with a permutation test (function permutest, 999 permutations) [47]. To ensure that PERMANOVA results were robust despite heterogenous multivariate dispersion [46–48], we produced 2D principal component analysis (PCA) ordination plots using ggvegan, ggordiplots, and export [49–51]. Third, we tested whether biofilms from lakes belonging to one group were more dispersed on average than biofilms from lakes of the other group. We used LMM with sample distance to its lake centroid as response variable, the grouping variable as fixed effect, and lake as random effect. Finally, we examined whether both groups were characterized by differentially abundant taxa using “analysis of compositions of microbiomes with bias correction” (ANCOM-BC), a method with low false discovery rates and high power [52, 53]. We used the function ancombc2 of package ANCOM-BC [52] with the group variable as fixed effect and lake as random effect, on all taxonomic levels (from ASV to class) of both the prokaryotic and micro-eukaryotic datasets. The false discovery rate correction was used to adjust *P*-values for multiple comparisons.

### Laboratory experiments

#### Harvesting and counting *Batrachochytrium dendrobatidis* zoospores

We used, for all experiments, the isolate IA043 of Bd-GPL (Global Panzootic Lineage; National Center for Biotechnology Information [NCBI] accession number: PRJNA413876, BioSample: SAMN07773623; name: IA043_cryo [54]), obtained from a recently metamorphosed individual of *A. obstetricans,* found dead in Acherito, Pyrenees, in 2004, and kindly provided by M. C. Fisher. All manipulations were performed under a laminar flow hood. This isolate was maintained in a 1% tryptone/0.2% glucose liquid medium by serial passage approximately every week, at a temperature of 19°C. After 1 week of development, ~3 ml of liquid cultures were deposited on agar gels (1% tryptone, 0.32% glucose, 1% agar). After 5–6 days at 19°C, we used 1–3 ml of mineral water (Volvic) to rinse the surface of the agar gels, waited 30 s, and filtered the supernatant with a 10 μm mesh to only collect zoospores. The concentration of the resulting solution (hereafter, zoospore solution) was assessed by averaging four counts on a Thoma hemocytometer under light microscopy (×100 magnification). For each count, a volume of 13 μl was introduced in the chamber, and all motile zoospores were counted in all 16 squares of the hemocytometer in a manner similar to all observers. Only motile zoospores were counted [55]. The zoospore solution was used to introduce a known number of Bd zoospores into the containers of our different experimental settings, which contained different kinds of biofilms. In all cases, we measured zoospore concentrations at t + 0 h (time of zoospore introduction; across all treatments, zoospore concentration at t + 0 h was in average 60.5 zoospores/0.1 μl, SD = 23.7), and then regularly for a total of four more times, in general at t + 1 h, 3 h, 6 h and 23 h (this timing was chosen based on preliminary experiments because no zoospore was ever seen swimming after 30 h) to estimate the rate at which they disappear from the water column (attachment to proximate surfaces, potentially including biofilms, or death). At each time point, we took four water samples at random places above the biofilm (a layer ~6.3 and 3.2 mm high when wells and Petri dishes were filled, respectively) and averaged the counts.

#### Natural and semi-natural biofilms

We first exposed zoospores to natural mountain biofilms: four 2 × 2 cm ceramic tiles (Casa Bella ref 8016609529738, all tiles have the same composition) were immersed in Gourg de Rabas during summer 2021, a Pyrenean alpine lake (2400 m above see level). Prior to deployment, tiles were brushed with soap and water, thoroughly rinsed, and then autoclaved. Gourg de Rabas biofilms (Fig. S2) contain potential Bd consumers such as rotifers and ciliates [31], and we observed ciliates during our experiments in the hemocytometer counting chamber. A prior study (unpublished data) showed that the composition of biofilms growing on artificial mineral substrates were quite similar to that of natural rock biofilms growing in the same lake as compared to a biofilm of another lake (Figs. S3 and S4), but the return to the laboratory (change in abiotic conditions) might have damaged the biofilm. The tiles (*n* = 4) were retrieved in summer 2022, transported back to the laboratory under stable temperature conditions (4–6°C), then individually placed in 35-mm-diameter Petri dishes (BD Falcon 351 008), and covered with 3 ml of zoospore solution, marking the beginning of the experiment. Controls (*n* = 2) consisted of a clean tile (no biofilm) with 3 ml of zoospore solution. During the short period of biofilm exposure (<48 h), zoospore concentrations followed an exponential decay law, as shown in another study [55], of which the disappearance rate from the water column, denoted λ (see Supplementary information for mathematical details), could be estimated with nonlinear modeling using the R package nlme [56]. Data from this and all kinetic experiments (i.e. natural, semi-natural, and simple artificial biofilms) were all fitted at once in a single model. The λ of each treatment (λ_biofilm_ or contrast) was compared with that of its controls (λ_control_), using emmeans [57]. Then, we corrected each λ_biofilm_ by its control to obtain the weighed (or net) zoospore disappearance rate of each treatment (i.e. λ_weighed_ = λ_biofilm_ - λ_control_) and compare them 2 × 2, adjusting for multiple comparison with the Šidák correction [58]. Because we fitted all our data in a single model, the same number of degrees of freedom (df = 313) was used by emmeans for all two-sided *t*-tests, i.e. for all *P*-value estimation of each contrast.

To produce a semi-natural biofilm, we placed tiles in the laboratory in a five liter boxes filled with dechlorinated tap water containing, in a 500 μm mesh, 10 g of shredded dead oak leaves. The decomposing leaves were sampled on soil in a grove outside the laboratory in July 2022 (43° 33′ 27.705″N, 1° 34′ 12.324″E). The leaves inoculated the water with organic nutrients and a variety of microorganisms likely including both prokaryotes and micro-eukaryotes. The presence of Bd-consuming micro-eukaryotes was possible although considered less likely than in the previous experiment. After 3 weeks, tiles were covered with biofilm and placed individually in Petri dishes and filled with 3 ml of zoospore solution. We had eight replicates with “leaf-shreds” biofilm and eight controls (i.e. clean tile with Volvic water).

#### Simple artificial biofilms

We grew artificial biofilms that did not contain zoospore consumers, produced in six-well plates (Corning Costar 3506) by introducing only one of the following phototrophic organisms: *N. palea*, *M. permitis* (both diatoms), and *Leptolyngbya* sp. (a cyanobacterium). Cyanobacteria and diatoms are building blocks of mountain lake biofilms (Fig. S2). These particular strains were selected as they quickly grow a biofilm under lab conditions. Each was maintained separately in a non-axenic bank at 19°C with a photoperiod of 16 h light/8 h dark (light intensity: 30–40 μmol.s^−1^.m^−2^), in the nutritive medium COMBO for diatoms and BG11 for the cyanobacterium [59, 60]. We harvested each strain separately in a 50 ml sterile Falcon tube, vortexed for 5 min to break cell aggregates. For diatoms, cell concentration was estimated with a Malassez counting chamber under an optical microscope (×100). In each well, we introduced 6 ml of *M. permitis* (5 × 10^6^ cells/ml), or six ml of *N. palea* (2.5 × 10^6^ cells/ml, as *N. palea* is roughly twice as large as *M. permitis*). For *Leptolyngbya* sp., we used a highly-efficient disperser (Ultra-Turax T25, Ika, Staufen, Germany) during 1 min. Recalcitrant aggregates were manually removed and cell concentration was then estimated with a spectrophotometer (wavelength of 663 nm) to obtain 6 ml of a solution with an absorbance between 0.290 and 0.295. Biofilms were left to grow for 7 days under constant temperature (19°C) and light intensity (30–40 μmol.s^−1^.m^−2^). We used two six-well plates per treatment with three biofilm wells and three control wells each (i.e. six replicates vs. six controls). Controls consisted of 6 ml of the medium used to grow the phototrophs. Three ml of zoospore solution were added to each well after removal of 3 ml of medium to keep the volume constant.

We tested whether the species richness of artificial biofilms could impact zoospore disappearance. It was conducted as in the previous experiment (six replicates vs. six controls spread in two six-well plates) except that in each well were introduced 6 ml of a solution containing the three phototrophs. The mix solution was made by adding the same volume of *M. permitis* solution (5 × 10^6^ cells/ml), *N. palea* solution (2.5 × 10^6^ cells/ml), and *Leptolyngbya* sp. solution (absorbance of 0.290–0.295 at 663 nm after diluting six times).

#### Survival of zoospores

To test whether zoospores were only attached but still viable, or truly inactivated following biofilm exposure (kinetic experiments), we exposed zoospores to *Leptolyngbya* biofilms as above, for a total of 15 wells containing a biofilm and 15 control wells. After 48 h in the six-well plate, we swabbed the walls and bottom of each well (one swab per well) where Bd zoospores were potentially attached. No zoospores are motile after a 48 h exposition. Thus, they can be attached and alive, or attached but dead, or dead in solution, but cannot be immotile but alive in solution [55]. Each swab tip (cellulose filaments; MW100, Medical & Wire Equipment Co, Essex, UK) was placed in distinct flasks containing 50 ml of typical liquid Bd growth medium to which we added antibiotics (200 mg/l penicillin G and 400 mg/l streptomycin, GIBCO Pen Strep [15]). Presence of Bd zoosporangia was assessed with inverted light microscopy (×100–200) after 7, 14, and 21 days. If no Bd zoosporangia/zoospores was observed by the end of this period, we considered that zoospores were inactivated following exposure. An “N -1” chi-squared test was used to analyze the results [61].

## Results

### Comparing biofilms from lakes with enzootic versus epizootic *Batrachochytrium dendrobatidis* infection dynamics

**Figure 1:**
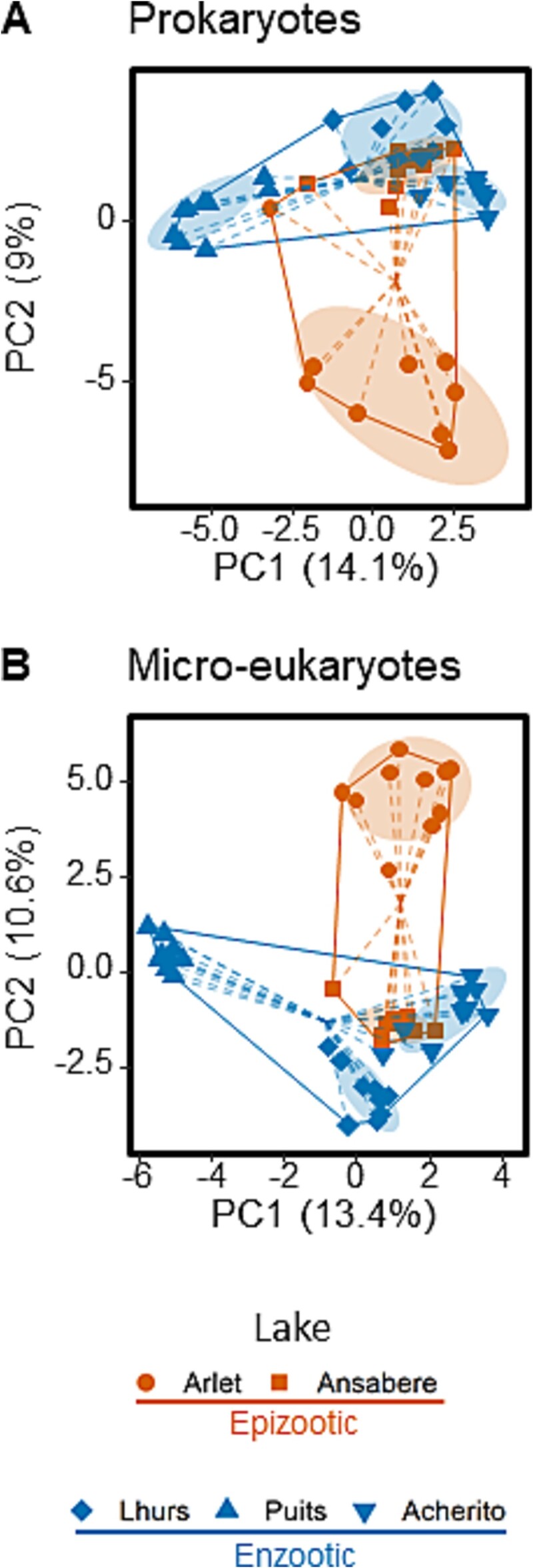
Different microbial composition of biofilms (*n* = 46) from lakes with enzootic (blue, *n* = 9 for the three lakes) versus epizootic (orange, *n* = 9 for Ansabere and 10 for Arlet) Bd infection dynamics. The first two axes of PCA ordinations (eigenvalues indicated) of prokaryotic (A) and micro-eukaryotic (B) biofilm assemblages on clr-transformed data are displayed. Points represent different samples and solid lines the hull of each group. Dotted lines represent the distance to the group centroid, indicating group β-dispersion. Filled ellipses contain 75% of the data for each lake and indicate intra-lake β-dispersion.

**Figure 2:**
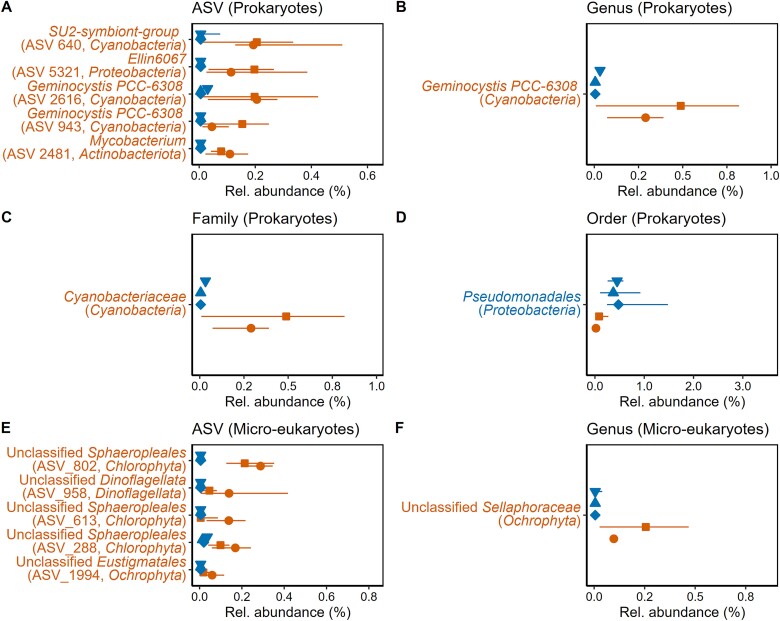
Taxa found as differentially abundant by ANCOM-BC between the enzootic (3 lakes, *n* = 9 for each lake) and epizootic lakes (2 lakes, *n* = 9 for Ansabere and 10 for Arlet) at different taxonomic resolution (A to F). Points indicate the median relative abundance of each taxa for each lake, and the bars indicate the interquartile range. Taxa are colored according to the group for which they are discriminative.

We compared the composition of biofilms (*n* = 46) sampled in summer from 2016 to 2020 from five geographically clustered lakes where amphibians have been continuously infected by Bd since 2004, but either with enzootic (three lakes) or epizootic dynamics (two lakes). Biofilm compositions significantly differed between groups, both for prokaryotic and micro-eukaryotic assemblages (PERMANOVA, respectively, *F*_1_ = 3.0 and 3.7, *R*^2^ = 0.63 and 0.78 with *P* < .001 for both), although lake effects seem more important than epizootic vs. enzootic effects to drive compositional variation (Figs 1and S3). We observed a significant heterogeneity in multivariate group dispersions in micro-eukaryotic assemblages (enzootic lakes more dispersed, *F*_1_ = 6.9, *P* = .013), but not in prokaryotic assemblages (*F*_1_ = 0.8, *P* = .389). Intra-lake biofilm dispersion was not significantly different between groups for both prokaryotes (*t*_3.03_ = 1.2, *P* = .301) and micro-eukaryotes (*t*_2.99_ = −0.3, *P* = .769), nor was α-diversity (Chao1 index; for prokaryotes, *t*_3.01_ = 0.4 and *P* = .747; for micro-eukaryotes, *t*_3.02_ = 0.4 and *P* = .694). However, several taxa were differentially abundant between groups (Fig. 2). The prokaryotic order *Pseudomonadales* was more abundant in enzootic lake biofilms. All other differentially abundant taxa were found to be discriminative of the epizootic lake biofilms, including the family *Cyanobacteriaceae* (in particular, genus *Geminocystis*), taxa from the genera *Ellin6067* (*Proteobacteria*) and *Mycobacterium* (*Actinobacteriota*), as well as several unclassified micro-eukaryotic taxa (Fig. 2).

### Effects of natural and semi-natural biofilms

**Figure 3:**
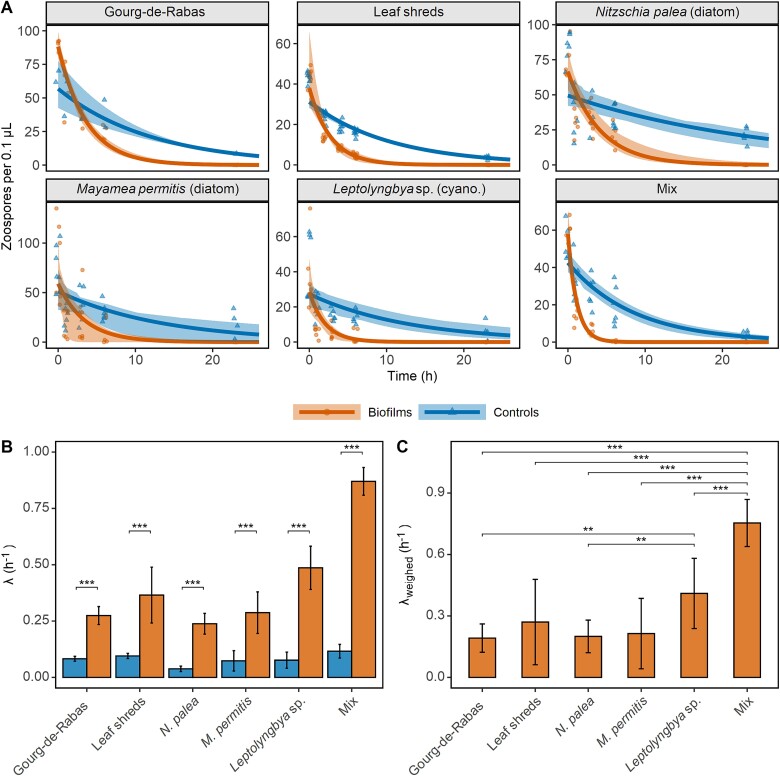
Zoospores disappear faster from the water column when exposed to biofilms. Evolution of zoospore concentration with time when exposed or not to different biofilms (A).Dots represent the data points (disks are for biofilms, triangles for controls), the solid lines are the fitted curves (exponential decay law, Z = Z_0_.E^-λt^), and shaded areas are the 95% confidence intervals around the fitted values. Uncorrected (B) and corrected (C; λ_weighed_ = λ_biofilm_ – λ_control_) zoospore disappearance rates for each treatment. Bars correspond to standard errors of the mean. The mix biofilm was made of *N. Palea, M. Permitis* and *Leptolyngbya* sp. Significance levels are displayed: *P* < .001 “^***^”, *P* < .01 “^**^”, *P* < .05 “^*^”, *P* > .0.5 “n.s.”. *P*-values were adjusted for multiple comparisons with the Šidák correction.

In the laboratory, we tested whether the presence of natural benthic biofilms, imported from Gourg de Rabas (a Pyrenean lake of which the *A. obstetricans* populations show signs of low infection burdens), could impact the number of motile zoospores over time, compared to a control without biofilm (four biofilms vs. two controls). Gourg de Rabas biofilms affected zoospores: their disappearance rate from the water column (λ) was significantly greater in the presence of biofilms than in their absence (*t*_313_ = 9.1, *P* < .001; Fig. 3A and B; Tables S2 and S3). We repeated the first experiment using semi-natural biofilms grown in the laboratory from shredded decomposing oak leaves (eight biofilms vs. eight controls). Zoospores disappeared significantly faster in the presence of the biofilms compared to controls (*t*_313_ = 4.3, *P* < .001; Fig. 3A and B), and the magnitude of the net biofilm effect λ_weighed_ was similar to that of the Gourg de Rabas biofilm (*t*_313_ = 1.2, ns, Fig. 3C).

### Effects of simple artificial biofilms

We used three types of simple artificially grown biofilm, not containing any known Bd zoospore consumers, produced either by the diatom *N. palea*, the diatom *M. permitis*, or the cyanobacterium *Leptolyngbya* sp. (all phototrophs, six biofilms vs. six controls each). In all three cases, Bd zoospores disappeared faster in the presence of the biofilm than in its absence (*t*_313_ = 8.3, 4.1, and 7.9 for *N. palea*, *M. permitis*, and *Leptolyngbya* sp, respectively, with *P* < .001 for all; Fig. 3A and B). The net effect of the cyanobacterium biofilm was significantly greater than those of *N. palea* and Gourg de Rabas biofilm (*t*_313_ = 3.7, *P* = 0.005 and *t*_313_ = 3.9 and *P* = .002, respectively; Fig. 3C). The biofilm made of the three phototrophs (*N. palea*, *M. permitis*, and *Leptolyngbya* sp.) not only had a greater disappearance rate than its control (six biofilms vs. six controls, *t*_313_ = 21.6, *P* < .001; Fig. 3A and B), but also the largest net effect, being significantly greater than those of all other biofilms considered in our study (*t*_313_ = 13.8, 6.7, 13.0, 8.6, and 5.5 against Gourg de Rabas, leaf shreds, *N. palea*, *M. permitis*, and *Leptolyngbya* sp biofilms, respectively, with *P* < .001 in all cases; Fig. 3C).

### Survival of zoospores

**Figure 4:**
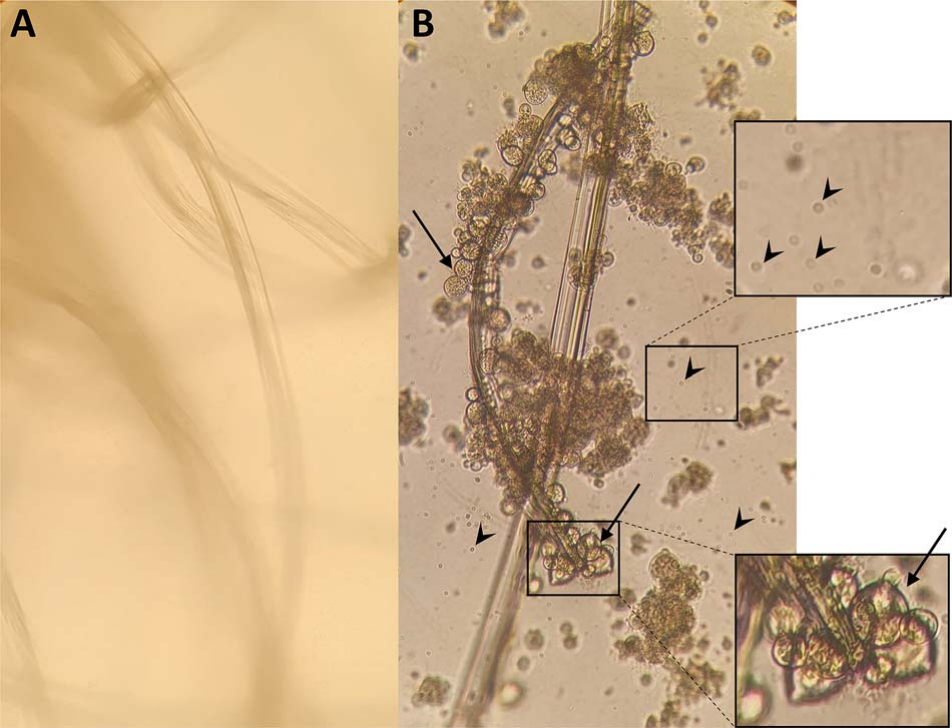
Photographs (400× magnification) of swab filaments placed in tryptone–glucose medium for 21 days, from swabs used to sample containers in which Bd zoospores were inoculated and left in the presence (A) or absence (B) of a biofilm for 48 h. In B and nearly all other controls (14 out of 15 flasks), many Bd zoosporangia (arrows) and motile zoospores (arrowheads) were visible, whereas nothing grew on swab filaments and flask walls of all cultures following biofilm exposure (A; 0/15).

We exposed Bd zoospores to *Leptolyngbya* sp. biofilms as in the previous experiment, then swabbed well walls and bottoms, and introduced the swab tips into the Bd growth medium with antibiotics to test whether zoospores were inactivated or still alive. Growth of Bd never resumed during the 21 days of monitoring (0/15) whereas, when not exposed to biofilms (controls), growth resumed in 93.3% of cases (14 out 15 flasks). Biofilm exposure significantly reduced Bd zoospore survival (*P* < .001, ${\chi}_1^2$ = 25.4; Fig. 4).

## Discussion

Here, we established that variation in biofilm composition was associated in natura with diverging infection dynamics of Bd*.* With our experimental series, we clearly demonstrated that biofilms negatively impacted the motility period and survival of the free-living infective stage of Bd, the zoospore. That biofilms inactivate Bd zoospores in a matter of hours could have major epidemiological implications, because (i) tadpoles feed on biofilms and (ii) Bd infects their mouthparts. If biofilms did not inactivate zoospores, the latter would attach on biofilms. Those attached zoospores would stay viable, as in our controls, for several weeks, if not months [20, 62], and hence could infect tadpoles during foraging. Because biofilms grow on all immersed surfaces in freshwater ecosystems, our results suggest that biofilms could limit the spread of Bd infection by reducing environmental transmission, which may in turn decrease host parasite burden, known to correlate with negative disease outcome at both the individual and populational levels [32]. Biofilms could thus constitute a biological barrier which contributes to protecting aquatic amphibians from Bd infection and chytridiomycosis by limiting environmental transmission. Our experiments revealed that different biofilms are not equivalent in their ability to inactivate zoospores, which is consistent with our observations in Pyrenean mountain lakes, where different biofilm community compositions coincided with divergent Bd infection dynamics.

The artificially grown biofilm formed by multiple phototrophic species had a greater effect on zoospores than all other tested biofilms, including those produced by the same phototrophic organisms individually. Increased biofilm biodiversity may therefore reduce risks of infectious diseases [63], and the rapid loss and changes in biofilm biodiversity recently observed in mountain lake ecosystems, where chytridiomycosis impacts are strong, would prove to be even more concerning [31]. We did not measure biofilm biomass, but future studies should do so, for instance by using crystal violet [22, 64], because biomass could be positively correlated to the inactivation effect strength (more biomass may mean more nutrient depletion, physicochemical entrapment, and/or secretion of allelopathic compounds). Consistent with this, artificial biofilms appeared visually thicker than natural and semi-natural biofilms and had greater impacts on zoospores. Natural and semi-natural biofilms may have compensated their low biomass by having Bd consumers, but both our experiments and our field data suggest that other mechanisms than zoospore consumption were at play because no Bd consumers were found in significantly different abundance in the field study, and even biofilms made of only one phototrophic organism had significant effects on zoospores. Other mechanism(s) may include nutrient depletion, known to decrease the period during which zoospores can survive and be infective [20]. Biofilms could also negatively affect the movement of zoospores by physicochemical interference of the matrix, or secrete molecules with allelopathic effects inducing either zoospore attachment or death. The two latter mechanisms would explain why *Leptolyngbya* sp. biofilms had a greater impact than other diatoms. *Leptolyngbya* is known to produce filaments that could physically interfere with the movement of zoospores. Further, species of that genus can also produce cyanotoxins harmful to other organisms, unlike diatoms [65, 66]. Chemical effects were supported by our in situ analyses, with *Pseudomonadales* found in significantly higher abundance in biofilms from lakes with enzootic disease dynamics. Several members of this order are inhibitory to Bd in culture and negatively associated with Bd infection on the skin of montane amphibians [67, 68].

All the putative mechanisms by which biofilms could inactivate Bd zoospores are not mutually exclusive, and would explain why the multispecies artificial biofilm had more effects on zoospores than single-species biofilms. Multispecies biofilms can exhibit emergent properties, i.e. properties that cannot be explained by its single components, such as increased nutrient sorption and transport [69]. Increased diversity in biofilms has been shown to increase biofilm efficiency in removing and degrading organic and inorganic nutrients, as well as chemicals, from the water column [70, 71]. The artificial multispecies biofilm may have been more efficient at trapping and using nutrients in our experiments, because it contained a higher diversity of species and/or because it may have achieved a higher biomass than mono-species biofilms (microbial richness and biomass are often positively correlated [22, 72, 73]). As a result, this biofilm may have depleted the aquatic environment of nutrients, and/or produced toxic molecules or intertwined structures such as filaments, to a greater extent than single-species biofilms did [20]. Under natural conditions, biofilms may influence Bd infection dynamics and outcome through ways other than zoospore inactivation, including effects on the host (e.g. through nutrition) and its microbiomes [7]. In our study, genera *Ellin6067* and *Mycobacterium* were found more abundant in biofilms of lakes with epizootic dynamics. Mycobacteria are known to cause disease to many species of amphibians and co-infections are acknowledged as possible drivers of other diseases [74, 75]. Also, *Ellin6067* is indicative of cyanobacterial blooms or xenobiotic pollution, which both could deteriorate amphibian health and thus promote chytridiomycosis outbreaks [76].

The antagonistic properties of biofilms toward Bd zoospores could be leveraged to develop biofilm-based tools and technologies, which could represent a potential avenue for in situ mitigation strategies. Existing strategies to mitigate chytridiomycosis in nature have considerable shortcomings from legal and ethical standpoints and are usually impractical [77]. Yet, biofilm approaches, such as biofilters or artificial aquatic mats, are nature-based, ecofriendly solutions, which are already widely used to remedy chemical and nutrient pollutions and remove waterborne pathogens [12, 13, 78]. More generally, our study suggests that biofilms contribute to the resistance and resilience of ecosystems against introduced microorganisms which may become invasive and/or, in the case of pathogenic microorganisms, cause the emergence or re-emergence of infectious diseases. For example, *Schistosoma* spp. also have a free-living aquatic stage. This pathogen is responsible for the zoonotic disease schistosomiasis, lethal to humans and endemic in Africa but emerging or reemerging in other continents including Europe or Asia [79, 80].

Previous work revealed that host-associated biofilms could prevent establishment of Bd, a major driver of biodiversity loss [22]; here, we show that biofilms associated with non-living surfaces (environmental biofilms) also inhibit Bd, highlighting the existence of antagonistic interactions that remain to be determined. Experimentally, we demonstrated that biofilms inactivated the infective stage of this zoosporic fungus, and that biofilm composition is an important factor in the strength of these effects. Thus, variations in biofilm composition in natura could explain the site-specific component in Bd prevalence and chytridiomycosis-related declines observed in some ecosystems such as the Pyrenees and the Sierra Nevada [25, 32]. We anticipate our study to be a starting point for more complex investigations on the interactions of biofilms with regard to Bd and how these roles are impacted by global change factors such as warming or pollution. For instance, zoospores could be exposed to only the liquid in which the biofilm grew, but without the biofilm, to test for the existence of metabolic allelopathic compounds secreted by biofilm dwellers. Nutrient depletion could also be tested by measuring nutrient concentrations at various times, and studying zoospore long-term survival in solutions of different nutrient concentrations. Physicochemical interference could be observed with scanning electron microscopy. Our findings promote the importance of including biotic environmental components in holistic health approaches. Not only would this improve our understanding of the eco-epidemiology of diseases, but it could also maximize the success of conservation practices, for example by selecting the most appropriate sites for reintroductions, and help finding novel nature-based solutions. Finally, our study also hints that ecological disruption and biodiversity loss augment the vulnerability of ecosystems to microbial invasions and emerging infectious diseases, causing further damage to our global life support system.

## Supplementary Material

ISMEJ-D-24-00817_Supplementary_information_R2_clean_wrae189

## Data Availability

Data and all codes (field and laboratory studies) for this paper are available on the Figshare repository at https://10.6084/m9.figshare.252367512. Biofilm sequence read data are archived in the European Nucleotide Archive (ENA) of the European Bioinformatics Institute (EBML-EBI) under the accession numbers PRJEB64636 (prokaryotes) and PRJEB65851 (eukaryotes). The sequence of the isolate Bd GPL IA043 was deposited in the NCBI Sequence Read Archive (SRA) under the accession number PRJNA413876 (BioSample: SAMN07773623; Sample name: IA043_cryo; SRA: SRS2757170).

## References

[1] Daszak P , Cunningham AA, Hyatt DA. Emerging infectious diseases of wildlife—threats to biodiversity and human health. *Science* 2000;287:443–9. 10.1126/science.287.5452.443 10642539

[2] Fisher MC , Henk DA, Briggs CJ et al. Emerging fungal threats to animal, plant and ecosystem health. *Nature* 2012;484:186–94. 10.1038/nature10947 22498624 PMC3821985

[3] Plowright RK , Sokolow SH, Gorman ME et al. Causal inference in disease ecology: investigating ecological drivers of disease emergence. *Front Ecol Environ* 2008;6:420–9. 10.1890/070086

[4] Thieltges DW , Jensen KT, Poulin R. The role of biotic factors in the transmission of free-living endohelminth stages. *Parasitology* 2008;135:407–26. 10.1017/S0031182007000248 18208633

[5] Johnson PTJ , Dobson A, Lafferty KD et al. When parasites become prey: ecological and epidemiological significance of eating parasites. *Trends Ecol Evol* 2010;25:362–71. 10.1016/j.tree.2010.01.005 20185202

[6] Flemming H-C , Wuertz S. Bacteria and archaea on earth and their abundance in biofilms. *Nat Rev Microbiol* 2019;17:247–60. 10.1038/s41579-019-0158-9 30760902

[7] Sentenac H , Loyau A, Leflaive J et al. The significance of biofilms to human, animal, plant and ecosystem health. *Funct Ecol* 2022;36:294–313. 10.1111/1365-2435.13947

[8] Hall-Stoodley L , Stoodley P. Biofilm formation and dispersal and the transmission of human pathogens. *Trends Microbiol* 2005;13:7–10. 10.1016/j.tim.2004.11.004 15639625

[9] Wingender J , Flemming H-C. Biofilms in drinking water and their role as reservoir for pathogens. *Int J Hyg Environ Health* 2011;214:417–23. 10.1016/j.ijheh.2011.05.009 21697011

[10] Chabaud S , Andres Y, Lakel A et al. Bacteria removal in septic effluent: influence of biofilm and protozoa. *Water Res* 2006;40:3109–14. 10.1016/j.watres.2006.06.008 16899272

[11] Rendueles O , Ghigo J-M. Multi-species biofilms: how to avoid unfriendly neighbors. *FEMS Microbiol Rev* 2012;36:972–89. 10.1111/j.1574-6976.2012.00328.x 22273363

[12] Maurya A , Singh MK, Kumar S. Biofiltration technique for removal of waterborne pathogens. In: Prasad MNV, Grobelak A (eds.), *Waterborne Pathogens: detection and treatments.* Butterworth-Heinemann, 1st edition. 2020;123–41. 10.1016/B978-0-12-818783-8.00007-4

[13] Steven JAC , Thorn RMS, Robinson GM et al. The control of waterborne pathogenic bacteria in fresh water using a biologically active filter. *Npj clean*. *Water* 2022;5:30–40. 10.1038/s41545-022-00169-y

[14] Scheele BC , Pasmans F, Skerratt LF et al. Amphibian fungal panzootic causes catastrophic and ongoing loss of biodiversity. *Science* 2019;363:1459–63. 10.1126/science.aav0379 30923224

[15] Longcore JE , Pessier AP, Nichols DK. *Batrachochytrium dendrobatidis* gen. Et sp. nov., a chytrid pathogenic to amphibians. *Mycologia* 1999;91:219–27. 10.1080/00275514.1999.12061011

[16] Berger L , Speare R, Daszak P et al. Chytridiomycosis causes amphibian mortality associated with population declines in the rain forests of Australia and central America. *Proc Natl Acad Sci* 1998;95:9031–6. 10.1073/pnas.95.15.9031 9671799 PMC21197

[17] Moss AS , Reddy NS, Dortaj IM et al. Chemotaxis of the amphibian pathogen *Batrachochytrium dendrobatidis* and its response to a variety of attractants. *Mycologia* 2008;100:1–5. 10.1080/15572536.2008.11832493 18488347

[18] Courtois EA , Loyau A, Bourgoin M et al. Initiation of *Batrachochytrium dendrobatidis* infection in the absence of physical contact with infected hosts—a field study in a high altitude lake. *Oikos* 2017;126:843–51. 10.1111/oik.03462

[19] Piotrowski JS , Annis SL, Longcore JE. Physiology of *Batrachochytrium dendrobatidis*, a chytrid pathogen of amphibians. *Mycologia* 2004;96:9–15. 10.1080/15572536.2005.11832990 21148822

[20] Johnson ML , Speare R. Survival of *Batrachochytrium dendrobatidis* in water: quarantine and disease control implications. *Emerg Infect Dis* 2003;9:922–5. 10.3201/eid0908.030145 12967488 PMC3020615

[21] Kearns PJ , Fischer S, Fernández-Beaskoetxea S et al. Fight fungi with fungi: antifungal properties of the amphibian mycobiome. *Front Microbiol* 2017;8:1–12. 10.3389/fmicb.2017.02494 29312201 PMC5735112

[22] Chen MY , Alexiev A, McKenzie VJ. Bacterial biofilm thickness and fungal inhibitory bacterial richness both prevent establishment of the amphibian fungal pathogen *Batrachochytrium dendrobatidis*. *Appl Environ Microbiol* 2022;88:e01604–21. 10.1128/aem.01604-21 35044804 PMC8904042

[23] Harris RN , Brucker RM, Walke JB et al. Skin microbes on frogs prevent morbidity and mortality caused by a lethal skin fungus. *ISME J* 2009;3:818–24. 10.1038/ismej.2009.27 19322245

[24] Searle CL , Mendelson JR, Green LE et al. Daphnia predation on the amphibian chytrid fungus and its impacts on disease risk in tadpoles. *Ecol Evol* 2013;3:4129–38. 10.1002/ece3.777 24324864 PMC3853558

[25] Schmeller DS , Blooi M, Martel A et al. Microscopic aquatic predators strongly affect infection dynamics of a globally emerged pathogen. *Curr Biol* 2014;24:176–80. 10.1016/j.cub.2013.11.032 24374305

[26] Altig R , Whiles MR, Taylor CL. What do tadpoles really eat? Assessing the trophic status of an understudied and imperiled group of consumers in freshwater habitats. *Freshw Biol* 2007;52:386–95. 10.1111/j.1365-2427.2006.01694.x

[27] Weitere M , Erken M, Majdi N et al. The food web perspective on aquatic biofilms. *Ecol Monogr* 2018;88:543–59. 10.1002/ecm.1315

[28] Mialet B , Majdi N, Tackx M et al. Selective feeding of bdelloid rotifers in river biofilms. *PLoS One* 2013;8:e75352. 10.1371/journal.pone.0075352 24073263 PMC3779155

[29] Walker SF , Bosch J, Gomez V et al. Factors driving pathogenicity vs. prevalence of amphibian panzootic chytridiomycosis in Iberia. *Ecol Lett* 2010;13:372–82. 10.1111/j.1461-0248.2009.01434.x 20132274

[30] Bates KA , Clare FC, O’Hanlon S et al. Amphibian chytridiomycosis outbreak dynamics are linked with host skin bacterial community structure. *Nat Commun* 2018;9:693. 10.1038/s41467-018-02967-w 29449565 PMC5814395

[31] Sentenac H , Loyau A, Zoccarato L et al. Biofilm community composition is changing in remote mountain lakes with a relative increase in potentially toxigenic algae. *Water Res* 2023;245:120547. 10.1016/j.watres.2023.120547 37708771

[32] Briggs CJ , Knapp RA, Vredenburg VT. Enzootic and epizootic dynamics of the chytrid fungal pathogen of amphibians. *Proc Natl Acad Sci* 2010;107:9695–700. 10.1073/pnas.0912886107 20457916 PMC2906864

[33] Klindworth A , Pruesse E, Schweer T et al. Evaluation of general 16S ribosomal RNA gene PCR primers for classical and next-generation sequencing-based diversity studies. *Nucleic Acids Res* 2013;41:e1. 10.1093/nar/gks808 22933715 PMC3592464

[34] Bradley IM , Pinto AJ, Guest JS. Design and evaluation of Illumina MiSeq-compatible, 18s rRNA gene-specific primers for improved characterization of mixed phototrophic communities. *Appl Environ Microbiol* 2016;82:5878–91. 10.1128/AEM.01630-16 27451454 PMC5038042

[35] Martin M . Cutadapt removes adapter sequences from high-throughput sequencing reads. *EMBnet J* 2011;17:10–2. 10.14806/ej.17.1.200

[36] R Core Team . R: A Language and Environment for Statistical Computing. 2022; https://www.R-project.org/

[37] Callahan BJ , McMurdie PJ, Rosen MJ et al. DADA2: high-resolution sample inference from Illumina amplicon data. *Nat Methods* 2016;13:581–3. 10.1038/nmeth.3869 27214047 PMC4927377

[38] Pruesse E , Peplies J, Glöckner FO. SINA: accurate high-throughput multiple sequence alignment of ribosomal RNA genes. *Bioinformatics* 2012;28:1823–9. 10.1093/bioinformatics/bts252 22556368 PMC3389763

[39] Quast C , Pruesse E, Yilmaz P et al. The SILVA ribosomal RNA gene database project: improved data processing and web-based tools. *Nucleic Acids Res* 2013;41:D590–6. 10.1093/nar/gks1219 23193283 PMC3531112

[40] McMurdie PJ , Holmes S. Phyloseq: an R package for reproducible interactive analysis and graphics of microbiome census data. *PLoS One* 2013;8:12. 10.1371/journal.pone.0061217 PMC3632530 23630581

[41] Gloor GB , Macklaim JM, Pawlowsky-Glahn V et al. Microbiome datasets are compositional: and this is not optional. *Front Microbiol* 2017;8:2224. 10.3389/fmicb.2017.02224 29187837 PMC5695134

[42] Aitchison J . The statistical analysis of compositional data. *J R Stat Soc Series B Stat Methodol* 1982;44:139–60. 10.1111/j.2517-6161.1982.tb01195.x

[43] Kuznetsova A , Brockhoff PB, Christensen RHB. Lmertest package: tests in linear mixed effects models. *J Stat Softw* 2017;82:1–26. 10.18637/jss.v082.i13

[44] Oksanen J , Simpson GL, Blanchet FG, et al. Vegan: Community Ecology Package. 2022; https://CRAN.R-project.org/package=vegan

[45] Chao A , Colwell RK, Lin C-W et al. Sufficient sampling for asymptotic minimum species richness estimators. *Ecology* 2009;90:1125–33. 10.1890/07-2147.1 19449706

[46] Anderson MJ . A new method for non-parametric multivariate analysis of variance. *Austral Ecol* 2001;26:32–46. 10.1111/j.1442-9993.2001.01070.pp.x

[47] Anderson MJ . Distance-based tests for homogeneity of multivariate dispersions. *Biometrics* 2006;62:245–53. 10.1111/j.1541-0420.2005.00440.x 16542252

[48] Schloss PD . Evaluating different approaches that test whether microbial communities have the same structure. *ISME J* 2008;2:265–75. 10.1038/ismej.2008.5 18239608

[49] Simpson GL . Ggvegan: “ggplot2” Plots for the “Vegan” Package. 2019; https://github.com/gavinsimpson/ggvegan

[50] Quensen J. Ggordiplots: Make Ggplot Versions of Vegan’s Ordiplots. 2021; http://github.com/jfq3/ggordiplots

[51] Wenseleers T , Vanderaa C. Export: Streamlined Export of Graphs and Data Tables. 2022; https://rdrr.io/github/tomwenseleers/export/

[52] Lin H , Peddada SD. Analysis of compositions of microbiomes with bias correction. *Nat Commun* 2020;11:3514. 10.1038/s41467-020-17041-7 32665548 PMC7360769

[53] Nearing JT , Douglas GM, Hayes MG et al. Microbiome differential abundance methods produce different results across 38 datasets. *Nat Commun* 2022;13:342. 10.1038/s41467-022-28034-z 35039521 PMC8763921

[54] O’Hanlon SJ , Rieux A, Farrer RA et al. Recent Asian origin of chytrid fungi causing global amphibian declines. *Science* 2018;360:621–7. 10.1126/science.aar1965 29748278 PMC6311102

[55] Woodhams DC , Alford RA, Briggs CJ et al. Life-history trade-offs influence disease in changing climates: strategies of an amphibian pathogen. *Ecology* 2008;89:1627–39. 10.1890/06-1842.1 18589527

[56] Pinheiro J , Bates D, R Core Team. Nlme: Linear and Nonlinear Mixed Effects Models. 2023; https://CRAN.R-project.org/package=nlme

[57] Lenth RV . Emmeans: Estimated Marginal Means, Aka Least-Squares Means. 2022; https://CRAN.R-project.org/package=emmeans

[58] Šidák Z . Rectangular confidence regions for the means of multivariate normal distributions. *J Am Stat Assoc* 1967;62:626–33. 10.1080/01621459.1967.10482935

[59] Stanier RY , Kunisawa R, Mandel M et al. Purification and properties of unicellular blue-green algae (order Chroococcales). *Bacteriol Rev* 1971;35:171–205. 10.1128/br.35.2.171-205.1971 4998365 PMC378380

[60] Kilham SS , Kreeger DA, Lynn SG et al. COMBO: a defined freshwater culture medium for algae and zooplankton. *Hydrobiologia* 1998;377:147–59. 10.1023/A:1003231628456

[61] Campbell I . Chi-squared and Fisher–Irwin tests of two-by-two tables with small sample recommendations. *Stat Med* 2007;26:3661–75. 10.1002/sim.2832 17315184

[62] Johnson ML , Speare R. Possible modes of dissemination of the amphibian chytrid *Batrachochytrium dendrobatidis* in the environment. *Dis Aquat Org* 2005;65:181–6. 10.3354/dao065181 16119886

[63] Keesing F , Belden LK, Daszak P et al. Impacts of biodiversity on the emergence and transmission of infectious diseases. *Nature* 2010;468:647–52. 10.1038/nature09575 21124449 PMC7094913

[64] Wilson C , Lukowicz R, Merchant S et al. Quantitative and qualitative assessment methods for biofilm growth: a mini-review. *Res Rev J Eng Technol* 2017;6. http://www.rroij.com/open-access/quantitative-and-qualitative-assessment-methods-for-biofilm-growth-a-minireview-.pdf PMC6133255 30214915

[65] Allen JL , Ten-Hage L, Leflaive J. Allelopathic interactions involving benthic phototrophic microorganisms. *Environ Microbiol Rep* 2016;8:752–62. 10.1111/1758-2229.12436 27337369

[66] Leflaive J , Ten-Hage L. Algal and cyanobacterial secondary metabolites in freshwaters: a comparison of allelopathic compounds and toxins. *Freshw Biol* 2007;52:199–214. 10.1111/j.1365-2427.2006.01689.x

[67] Kueneman JG , Weiss S, McKenzie VJ. Composition of micro-eukaryotes on the skin of the cascades frog (*Rana cascadae*) and patterns of correlation between skin microbes and *Batrachochytrium dendrobatidis*. *Front Microbiol* 2017;8:8. 10.3389/fmicb.2017.02350 29276502 PMC5727676

[68] Woodhams DC , Alford RA, Antwis RE et al. Antifungal isolates database of amphibian skin-associated bacteria and function against emerging fungal pathogens. *Ecology* 2015;96:595–5. 10.1890/14-1837.1

[69] Flemming H-C , Wingender J, Szewzyk U et al. Biofilms: an emergent form of bacterial life. *Nat Rev Microbiol* 2016;14:563–75. 10.1038/nrmicro.2016.94 27510863

[70] Cardinale BJ . Biodiversity improves water quality through niche partitioning. *Nature* 2011;472:86–9. 10.1038/nature09904 21475199

[71] Burmølle M , Ren D, Bjarnsholt T et al. Interactions in multispecies biofilms: do they actually matter? *Trends Microbiol* 2014;22:84–91. 10.1016/j.tim.2013.12.004 24440178

[72] Murga R , Stewart PS, Daly D. Quantitative analysis of biofilm thickness variability. *Biotechnol Bioeng* 1995;45:503–10. 10.1002/bit.260450607 18623250

[73] Wang C , Liu D, Bai E. Decreasing soil microbial diversity is associated with decreasing microbial biomass under nitrogen addition. *Soil Biol Biochem* 2018;120:126–33. 10.1016/j.soilbio.2018.02.003

[74] Chai N . Mycobacteriosis in amphibians. In: Miller R.E., Fowler M.E. (eds.), Fowler’s Zoo and Wild Animal Medicine: Current Therapy, Vol. 7. St. Louis: W.B. Saunders, 2011, 224–30.

[75] Herczeg D , Ujszegi J, Kásler A et al. Host–multiparasite interactions in amphibians: a review. *Parasit Vectors* 2021;14:296. 10.1186/s13071-021-04796-1 34082796 PMC8173923

[76] Lezcano MÁ , Velázquez D, Quesada A et al. Diversity and temporal shifts of the bacterial community associated with a toxic cyanobacterial bloom: an interplay between microcystin producers and degraders. *Water Res* 2017;125:52–61. 10.1016/j.watres.2017.08.025 28829999

[77] Garner TWJ , Schmidt BR, Martel A et al. Mitigating amphibian chytridiomycoses in nature. *Philos Trans R Soc B* 2016;371: 20160207. 10.1098/rstb.2016.0207 PMC5095549 28080996

[78] Sonawane JM , Rai AK, Sharma M et al. Microbial biofilms: recent advances and progress in environmental bioremediation. *Sci Total Environ* 2022;824:153843. 10.1016/j.scitotenv.2022.153843 35176385

[79] Kincaid-Smith J , Rey O, Toulza E et al. Emerging schistosomiasis in Europe: a need to quantify the risks. *Trends Parasitol* 2017;33:600–9. 10.1016/j.pt.2017.04.009 28539255

[80] Liang S , Yang C, Zhong B et al. Re-emerging schistosomiasis in hilly and mountainous areas of Sichuan. *China Bull World Health Organ* 2006;84:139–44. 10.2471/BLT.05.025031. https://www.scielosp.org/pdf/bwho/v84n2/v84n2a15.pdf 16501732 PMC2626530

