# Supplementary material for: Biofilms inactivate the free-living stage of Batrachochytrium dendrobatidis, the most destructive pathogen for vertebrate diversity
Source: ISME J. 2024 Sep 26;18(1):wrae189. doi: 10.1093/ismejo/wrae189 (PMC11630259; doi:10.1093/ismejo/wrae189)
Supplement: ISMEJ-D-24-00817_Supplementary_information_R2_clean_wrae189 [file ismej-d-24-00817_supplementary_information_r2_clean_wrae189.docx]

**Supplementary information for:**

**Sentenac *et al.* (2024) `Biofilms inactivate the most destructive pathogen for vertebrate diversity` *The ISME Journal***

Authors
Hugo Sentenac^a,b,c^ *, Dirk S. Schmeller^a^, Solène Caubet^a^, Adélaïde Carsin^a^, Rémi Guillet^a^, Jessica Ferriol^a^, Joséphine Leflaive^a^, Adeline Loyau^a^

Affiliations
^a^ Centre de Recherche sur la Biodiversité et l’Environnement (CRBE, UMR 5300), Université de Toulouse, CNRS, IRD, Toulouse INP, Université Toulouse 3 - Paul Sabatier (UT3), 118 route de Narbonne, 31062 Toulouse Cedex 9, France
^b^ CBGP, Université de Montpellier, CIRAD, INRAE, Institut Agro, IRD, Avenue du Campus Agropolis, 34980 Montferrier-sur-Lez, France
^c^ Chrono-environnement UMR 6249 CNRS/UFC, Université de Franche-Comté, 16 route de Gray, 25 000 Besançon, France

*Corresponding author: [hugo.sentenac@univ-fcomte.fr](mailto:hugo.sentenac@univ-fcomte.fr)
Physical address: Laboratoire Chrono-Environnement UMR 6249, CNRS/UFC, Université de Franche-Comté – Bâtiment Propédeutique, 16 Route de Gray, F-25030 Besançon cedex, France

**This file includes**: Protocols for PCR amplification and purification, Determination of zoospore disappearance rate: mathematical details, Supplementary Tables S1, S2, S3, as well as Supplementary Figures S1, S2, S3, and S4, and finally, Supplementary references.

Protocols for PCR amplification and purification

Our PCR mix contained 12.5μL MyTaq (Bioline, Meridian Bioscience), 1μL of 1μM solution of both forward and reverse primer, 0.5μL of bovine serum albumin, and 10µL of diluted template to obtain exactly 20ng of DNA in each well. The PCR conditions were as follows: 95°C for 3 minutes, 30 cycles at 95°C for 30s, 55°C for 30s and 72°C for 30s, with a final extension step at 72°C for 5 minutes. The PCR products were sent to an independent research platform, GeT Biopuces (https://get-biopuces.insa-toulouse.fr/), which performed the indexing PCR and amplicon sequencing. Briefly, PCR products were cleaned using Agencourt AMPure XP (Thermofisher Scientific, France) and submitted to a 10-cycle PCR according to the standard Illumina protocol (95°C for 180s, 10 cycles x [95°C for 30s, 55°C for 30s, 72°C for 30s] and 72°C for 300s) with sample-specific Illumina-Nextera Index primers. Products were cleaned, quantified and diluted to include 140ng of DNA in the pool.

Determination of zoospore disappearance rate: mathematical details

During the short period of biofilm exposure (< 48h), only mortality and attachment on the sides of the wells or in the biofilms can affect zoospore concentration [1]. Therefore, the rate of change of the zoospore concentration can be described by:

$$\begin{aligned} \frac{dZ\left( t \right)}{dt}= -\left( m_{z}+f_{z} \right).Z\left( t \right).\#\left( 1a \right) \end{aligned}$$

Z(t) is the concentration of motile zoospores at time t, m_z_ is the mortality rate of zoospore and f_z_ their attachment rate. The zoospore disappearance rate λ can be defined as

$$\begin{aligned} \lambda= m_{z}+f_{z} \#\left( 2 \right) \end{aligned}$$

which means that equation (1) can be written:

$$\begin{aligned} \frac{dZ\left( t \right)}{dt}= -\lambda.Z\left( t \right).\#\left( 1b \right) \end{aligned}$$

One primitive function of equation 1b is

$$\begin{aligned} Z\left( t \right)=Z\left( 0 \right){.e}^{-\lambda.t}\#\left( 3 \right) \end{aligned}$$

Equation (3) is the classic formula describing an exponential decay law. We fitted a non-linear model to all our data at once to estimate the disappearance rate λ for each treatment as described in the main manuscript.

# Supplementary tables

## Table S1.

**Table S1**: **Description of the studied lakes and aquatic physico-chemical conditions at the moment of biofilm sampling.** Abbreviations: dO2: dissolved dioxygen; TOC: total organic carbon; TN total nitrogen; NO3: nitrate; water T denotes the mean water temperature during the week prior to sampling (T was recorded with in situ loggers). Colors indicate the range of values per column, i.E. per variable (dark green: lowest value, yellow: mean value; dark red: highest values)

| **Lake** | **latitude** | **longitude** | **altitude** | **lake_area** | **Date** | **pH** | **dO2 (mg/l)** | **Conductivity (µS/cm)** | **TOC (mg/l)** | **TN (mg/l)** | **Hardness (mg/l)** | **NO3 (mg/l)** | **water T (°C)** |
| --- | --- | --- | --- | --- | --- | --- | --- | --- | --- | --- | --- | --- | --- |
| Acherito | 42.879041 | -0.708854 | 1880 | 7.46 | 22/06/2016 | 8.30 | 10.33 | 111.38 | 6.92 | 0.22 | 20.97 | 0.05 | 10.91 |
|  |  |  |  |  | 20/09/2016 |  |  |  | 4.53 | 1.36 | 21.61 | 0.01 | 13.97 |
|  |  |  |  |  | 04/07/2017 | 8.76 |  |  |  |  | 18.76 | 0.02 | 15.00 |
|  |  |  |  |  | 14/08/2017 | 8.67 | 9.39 |  | 2.32 | 0.61 | 19.65 | 0.01 | 16.08 |
|  |  |  |  |  | 09/10/2017 |  |  |  | 4.70 | 1.04 | 18.84 | 0.00 | 12.11 |
|  |  |  |  |  | 03/07/2018 |  |  |  |  |  |  |  | 17.14 |
|  |  |  |  |  | 15/08/2018 |  |  |  |  |  | 26.55 | 0.03 | 16.66 |
|  |  |  |  |  | 27/09/2018 |  |  |  |  |  | 25.11 | 0.00 | 13.20 |
|  |  |  |  |  | 02/08/2019 |  |  |  |  |  | 21.11 | 0.01 | 15.12 |
| Ansabere | 42.8877799 | -0.7088499 | 1850 | 0.21 | 22/06/2016 | 8.38 | 10.76 | 86.23 | 3.20 | 0.81 | 13.49 | 0.06 | 8.09 |
|  |  |  |  |  | 20/09/2016 |  |  |  | 6.53 | 0.18 | 15.77 | 0.00 | 9.34 |
|  |  |  |  |  | 04/07/2017 | 8.47 |  |  | 1.43 | 0.30 | 11.99 | 0.03 | 11.62 |
|  |  |  |  |  | 14/08/2017 | 8.18 | 10.52 |  | 3.13 | 0.67 | 14.22 | 0.04 | 13.69 |
|  |  |  |  |  | 09/10/2017 |  |  |  | 6.53 | 0.17 | 14.75 | 0.04 | 8.33 |
|  |  |  |  |  | 03/07/2018 |  |  |  |  |  |  |  | 17.28 |
|  |  |  |  |  | 15/08/2018 |  |  |  |  |  | 18.31 | 0.00 | 16.81 |
|  |  |  |  |  | 27/09/2018 |  |  |  |  |  | 18.21 | 0.01 | 11.61 |
|  |  |  |  |  | 02/08/2019 |  |  |  |  |  | 14.99 | 0.02 | 15.96 |
|  |  |  |  |  | 02/09/2020 |  |  |  |  |  |  |  | 14.92 |
| Arlet | 42.8403799 | -0.6149999 | 1974 | 3.46 | 23/06/2016 | 9.21 | 10.08 | 50.80 | 3.95 | 1.19 | 12.76 | 0.00 | 12.02 |
|  |  |  |  |  | 21/09/2016 |  |  |  | 6.49 | 0.19 | 10.50 | 0.07 | 10.91 |
|  |  |  |  |  | 04/07/2017 |  |  |  | 2.36 | 0.76 | 9.45 | 0.00 | 14.09 |
|  |  |  |  |  | 15/08/2017 | 9.26 | 10.19 |  | 3.28 | 0.70 | 9.65 | 0.00 | 16.45 |
|  |  |  |  |  | 09/10/2017 |  |  |  | 3.98 | 0.67 | 9.71 | 0.00 | 11.76 |
|  |  |  |  |  | 03/07/2018 |  |  |  |  |  |  |  | 19.06 |
|  |  |  |  |  | 15/08/2018 |  |  |  |  |  | 12.01 | 0.00 | 17.77 |
|  |  |  |  |  | 27/09/2018 |  |  |  |  |  | 12.46 | 0.00 | 12.63 |
|  |  |  |  |  | 03/08/2019 |  |  |  |  |  | 9.06 | 0.00 | 18.27 |
|  |  |  |  |  | 03/09/2020 |  |  |  |  |  |  |  | 16.69 |
| Puits | 42.86403 | -0.63343 | 1880 | 0.26 | 23/06/2016 | 7.60 | 9.91 | 11.80 | 2.42 | 0.82 | 2.88 | 0.01 | 13.54 |
|  |  |  |  |  | 21/09/2016 |  |  |  | 6.48 | 0.22 | 3.11 | 0.01 | 10.68 |
|  |  |  |  |  | 04/07/2017 |  |  |  | 2.06 | 0.66 | 2.61 | 0.01 | 15.27 |
|  |  |  |  |  | 15/08/2017 | 7.82 | 9.61 |  | 4.17 | 1.36 | 2.56 | 0.01 | 16.26 |
|  |  |  |  |  | 09/10/2017 |  |  |  | 3.96 | 0.86 | 2.44 | 0.02 | 10.70 |
|  |  |  |  |  | 03/07/2018 |  |  |  |  |  |  |  | 19.32 |
|  |  |  |  |  | 15/08/2018 |  |  |  |  |  | 3.76 | 0.01 | 18.48 |
|  |  |  |  |  | 27/09/2018 |  |  |  |  |  | 3.94 | 0.01 | 13.93 |
|  |  |  |  |  | 03/09/2020 |  |  |  |  |  |  |  | 15.01 |
| Lhurs | 42.921616 | -0.703112 | 1697 | 3.59 | 21/06/2016 | 8.84 | 10.08 | 133.85 |  |  | 24.00 | 0.04 | 8.76 |
|  |  |  |  |  | 19/09/2016 |  |  |  | 6.73 | 0.20 | 28.49 | 0.10 | 11.25 |
|  |  |  |  |  | 03/07/2017 | 8.46 |  |  | 1.73 | 0.38 |  | 0.07 | 12.48 |
|  |  |  |  |  | 13/08/2017 | 8.72 | 9.72 |  | 2.97 | 0.75 | 23.93 | 0.01 | 15.34 |
|  |  |  |  |  | 08/10/2017 |  |  |  | 6.60 | 0.19 | 21.77 | 0.01 | 11.68 |
|  |  |  |  |  | 02/07/2018 |  |  |  |  |  |  |  | 17.82 |
|  |  |  |  |  | 14/08/2018 |  |  |  |  |  | 32.23 | 0.02 | 18.78 |
|  |  |  |  |  | 26/09/2018 |  |  |  |  |  | 26.95 | 0.00 | 14.87 |
|  |  |  |  |  | 04/08/2019 |  |  |  |  |  | 30.22 | 0.00 | 17.85 |
|  |  |  |  |  | 02/09/2020 |  |  |  |  |  |  |  | 14.21 |

## Table S2

**Table S2**: **Estimates of the zoospore disappearance constant λ of each treatment and its control, and of their respective difference (λ_weighed_ = λ_biofilm_ – λ_control_).** P values were adjusted for multiple comparisons using the Šidák correction. SE = Standard error, df = degrees of freedom, CI = 95% confidence intervals, adj. = adjusted.

| **Treatment** | **λ** | **SE** | **df** | **Lower CI** | **Upper CI** | ***t* ratio** | **Adj. *P* value** |
| --- | --- | --- | --- | --- | --- | --- | --- |
| Mix controls | 0.116 | 0.015 | 313 | 0.086 | 0.147 | 7.54 | < 0.001 |
| Mix biofilms | 0.870 | 0.031 | 313 | 0.808 | 0.932 | 27.8 | < 0.001 |
| *Leptolyngbya* sp. controls | 0.076 | 0.018 | 313 | 0.041 | 0.112 | 4.19 | < 0.001 |
| *Leptolyngbya* sp. biofilms | 0.486 | 0.049 | 313 | 0.390 | 0.582 | 9.97 | < 0.001 |
| *Mayamea permitis* controls | 0.073 | 0.023 | 313 | 0.028 | 0.119 | 3.19 | 0.002 |
| *Mayamea permitis* biofilms | 0.287 | 0.047 | 313 | 0.195 | 0.379 | 6.13 | < 0.001 |
| *Nitzschia palea* controls | 0.038 | 0.006 | 313 | 0.026 | 0.050 | 6.18 | < 0.001 |
| *Nitzschia palea* biofilms | 0.238 | 0.023 | 313 | 0.192 | 0.284 | 10.18 | < 0.001 |
| Gourg de Rabas controls | 0.083 | 0.006 | 313 | 0.072 | 0.093 | 14.9 | < 0.001 |
| Gourg de Rabas biofilms | 0.274 | 0.020 | 313 | 0.235 | 0.314 | 13.54 | < 0.001 |
| Leaf_shreds controls | 0.095 | 0.006 | 313 | 0.084 | 0.107 | 16.17 | < 0.001 |
| Leaf_shreds biofilms | 0.365 | 0.063 | 313 | 0.241 | 0.489 | 5.8 | < 0.001 |
| **Contrasts: biofilm - control** | **λ_weighed_** | **SE** | **df** | **Lower CI** | **Upper CI** | ***t* ratio** | **Adj. *P* value** |
| Mix | 0.754 | 0.035 | 313 | 0.661 | 0.846 | 21.6 | < 0.001 |
| *Leptolyngbya sp.* | 0.410 | 0.052 | 313 | 0.272 | 0.548 | 7.87 | < 0.001 |
| *Mayamea permitis* | 0.214 | 0.052 | 313 | 0.076 | 0.352 | 4.1 | < 0.001 |
| *Nitzschia palea* | 0.200 | 0.024 | 313 | 0.136 | 0.264 | 8.27 | < 0.001 |
| Gourg de Rabas | 0.192 | 0.021 | 313 | 0.136 | 0.247 | 9.13 | < 0.001 |
| Leaf shreds | 0.270 | 0.063 | 313 | 0.103 | 0.438 | 4.27 | < 0.001 |

## Table S3

**Table S3:** **Pairwise comparisons of the zoospore disappearance rates λ between controls (absence of biofilms) and λ_weighed_ (λ_biofilm_ – λ_control_) between biofilms**. P values were adjusted for multiple comparisons using the Šidák correction. SE = Standard error, df= degrees of freedom, CI = 95% confidence intervals, adj. = adjusted.

| **Control 1** | **Control 2** | **estimate** | **SE** | **df** | **Lower CI** | **Upper CI** | ***t* ratio** | **Adj. *P* value** |
| --- | --- | --- | --- | --- | --- | --- | --- | --- |
| Gourg de Rabas | Leaf_shreds | -0.013 | 0.008 | 313 | -0.036 | 0.011 | -1.56 | 0.854 |
| Gourg de Rabas | *Leptolyngbya sp.* | 0.006 | 0.019 | 313 | -0.05 | 0.062 | 0.33 | 1.000 |
| Gourg de Rabas | *M. permitis* | 0.009 | 0.024 | 313 | -0.061 | 0.079 | 0.38 | 1.000 |
| Gourg de Rabas | Mix | -0.034 | 0.016 | 313 | -0.082 | 0.015 | -2.06 | 0.463 |
| Gourg de Rabas | *N. palea* | 0.045 | 0.008 | 313 | 0.020 | 0.069 | 5.39 | < 0.001 |
| Leaf_shreds | *Leptolyngbya sp.* | 0.019 | 0.019 | 313 | -0.038 | 0.075 | 0.98 | 0.997 |
| Leaf_shreds | *M. permitis* | 0.022 | 0.024 | 313 | -0.048 | 0.092 | 0.91 | 0.999 |
| Leaf_shreds | Mix | -0.021 | 0.017 | 313 | -0.070 | 0.028 | -1.28 | 0.966 |
| Leaf_shreds | *N. palea* | 0.057 | 0.009 | 313 | 0.032 | 0.082 | 6.72 | < 0.001 |
| *Leptolyngbya sp.* | *M. permitis* | 0.003 | 0.029 | 313 | -0.084 | 0.089 | 0.10 | 1.000 |
| *Leptolyngbya sp.* | Mix | -0.040 | 0.024 | 313 | -0.110 | 0.030 | -1.67 | 0.778 |
| *Leptolyngbya sp.* | *N. palea* | 0.038 | 0.019 | 313 | -0.018 | 0.095 | 2.00 | 0.512 |
| *M. permitis* | Mix | -0.043 | 0.028 | 313 | -0.124 | 0.039 | -1.55 | 0.861 |
| *M. permitis* | *N. palea* | 0.035 | 0.024 | 313 | -0.035 | 0.106 | 1.49 | 0.891 |
| Mix | *N. palea* | 0.078 | 0.017 | 313 | 0.029 | 0.127 | 4.72 | < 0.001 |
| **Biofilm 1** | **Biofilm 2** | **estimate** | **SE** | **df** | **Lower CI** | **Upper CI** | ***t* ratio** | **Adj. P-value** |
| Gourg de Rabas | *Leaf_shreds* | 0.078 | 0.067 | 313 | -0.118 | 0.275 | 1.18 | 0.984 |
| Gourg de Rabas | *Leptolyngbya sp.* | 0.218 | 0.056 | 313 | 0.052 | 0.384 | 3.88 | 0.002 |
| Gourg de Rabas | *M. permitis* | 0.022 | 0.056 | 313 | -0.144 | 0.188 | 0.39 | 1.000 |
| Gourg de Rabas | *Mix* | 0.562 | 0.041 | 313 | 0.442 | 0.682 | 13.8 | < 0.001 |
| Gourg de Rabas | *N. palea* | 0.008 | 0.032 | 313 | -0.086 | 0.103 | 0.26 | 1.000 |
| Leaf_shreds | *Leptolyngbya sp.* | 0.140 | 0.082 | 313 | -0.102 | 0.381 | 1.71 | 0.753 |
| Leaf_shreds | *M. permitis* | -0.056 | 0.082 | 313 | -0.298 | 0.186 | -0.69 | 1.000 |
| Leaf_shreds | Mix | 0.484 | 0.072 | 313 | 0.270 | 0.697 | 6.69 | < 0.001 |
| Leaf_shreds | *N. palea* | -0.070 | 0.068 | 313 | -0.270 | 0.130 | -1.03 | 0.995 |
| *Leptolyngbya sp.* | *M. permitis* | -0.196 | 0.074 | 313 | -0.414 | 0.021 | -2.66 | 0.116 |
| *Leptolyngbya sp.* | *Mix* | 0.344 | 0.063 | 313 | 0.159 | 0.529 | 5.49 | < 0.001 |
| *Leptolyngbya sp.* | *N. palea* | -0.210 | 0.057 | 313 | -0.379 | -0.040 | -3.65 | 0.005 |
| *M. permitis* | Mix | 0.540 | 0.063 | 313 | 0.355 | 0.725 | 8.60 | < 0.001 |
| *M. permitis* | *N. palea* | -0.014 | 0.058 | 313 | -0.183 | 0.156 | -0.24 | 1.000 |
| Mix | *N. palea* | -0.554 | 0.042 | 313 | -0.679 | -0.428 | -13.0 | < 0.001 |

# Supplementary figures

## Figure S1


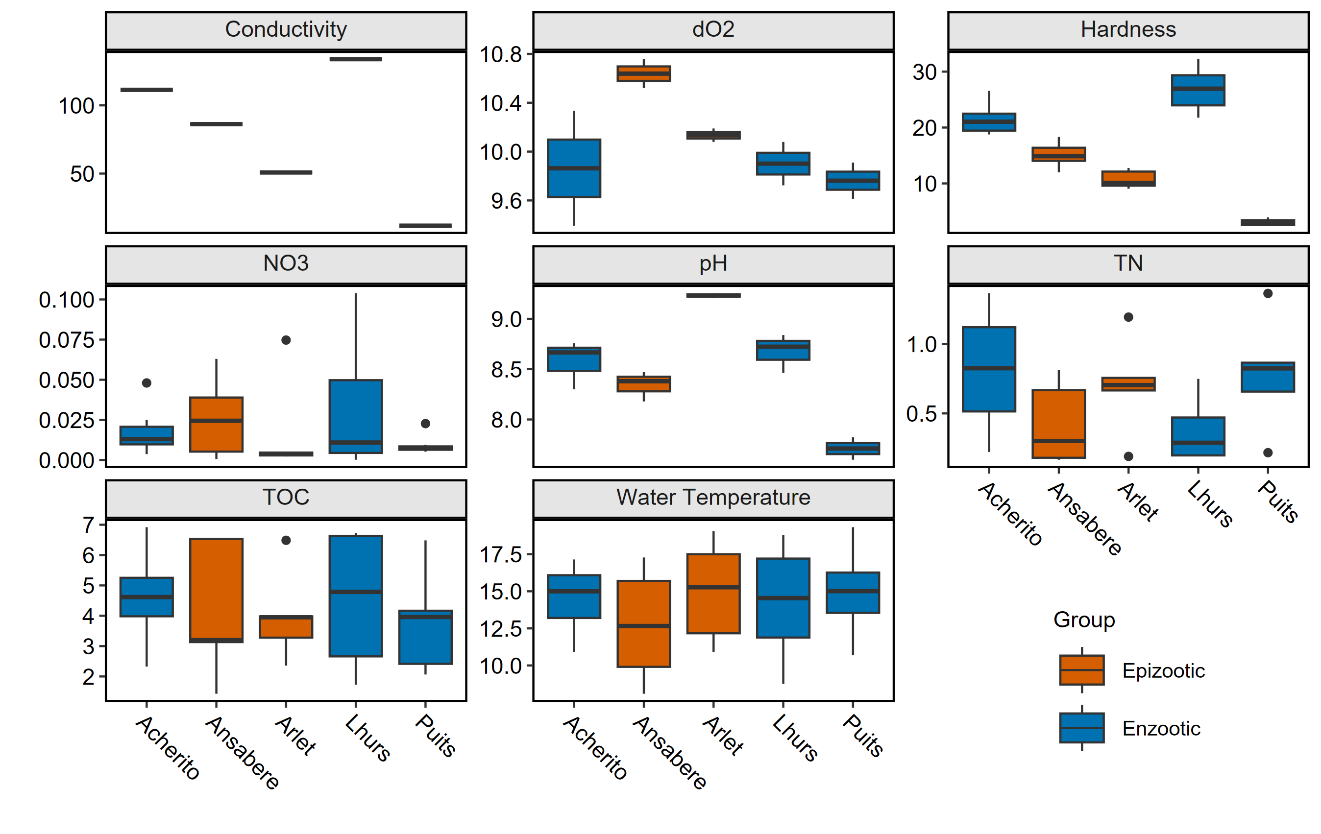


**Figure S1:** **Physico-chemical conditions at the time of biofilm sampling in the five studied lakes from 2016 to 2020 (see also Table S1)**. None of the physico-chemical variables correlate with the long-term epidemiology of Bd infections (group: enzootic or epizootic) in these lakes, hence why we study biotic environmental factors. Abbreviations and units: Conductivity in µS/cm, dO2: dissolved dioxygen (mg/l), Hardness (mg/l), NO3: nitrate (mg/l), pH: potential hydrogen, TN: total nitrogen (mg/l), TOC: total organic carbon (mg/l), water temperature: mean water temperature during the week prior to sampling (°C).

## Figure S2


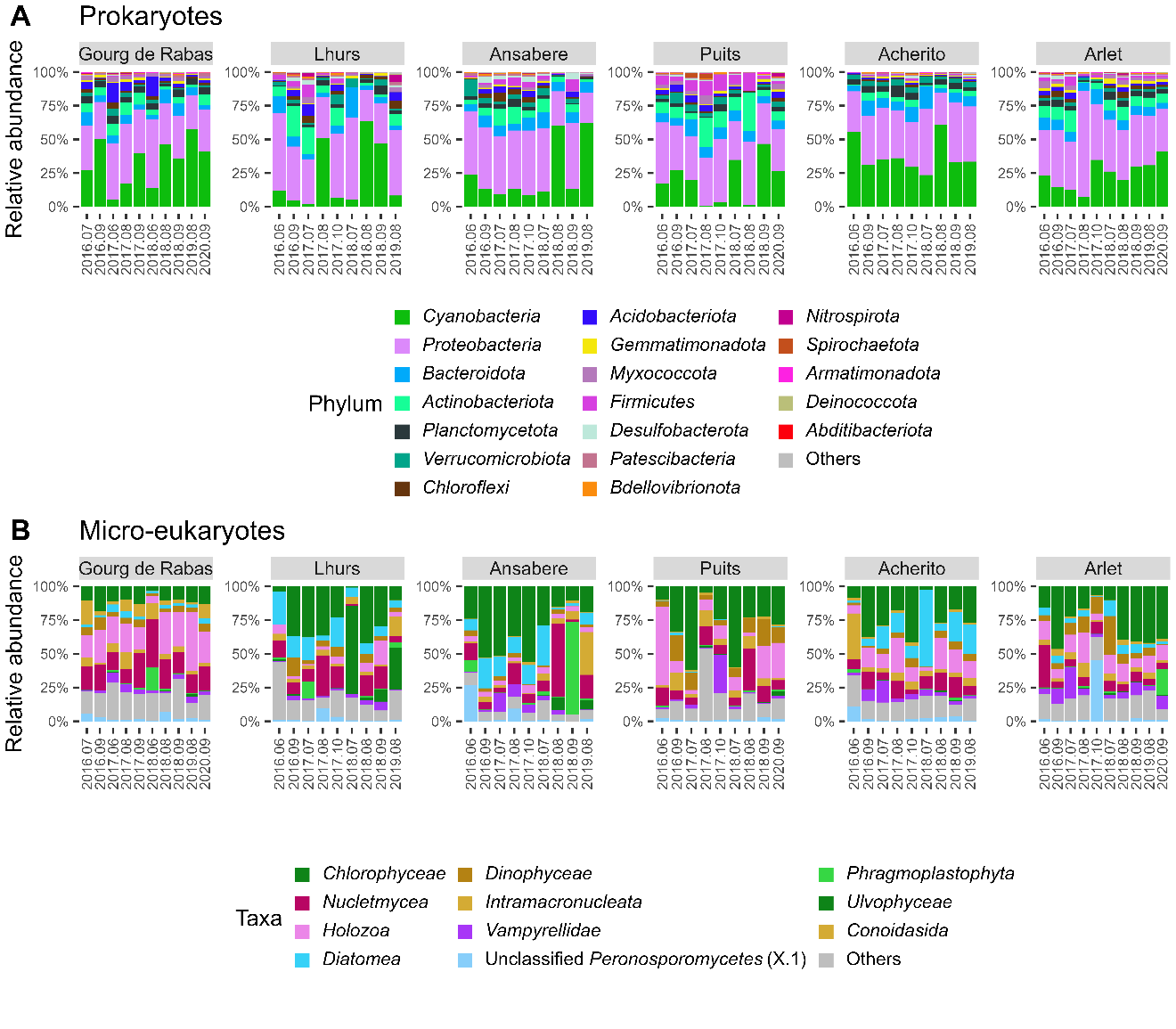

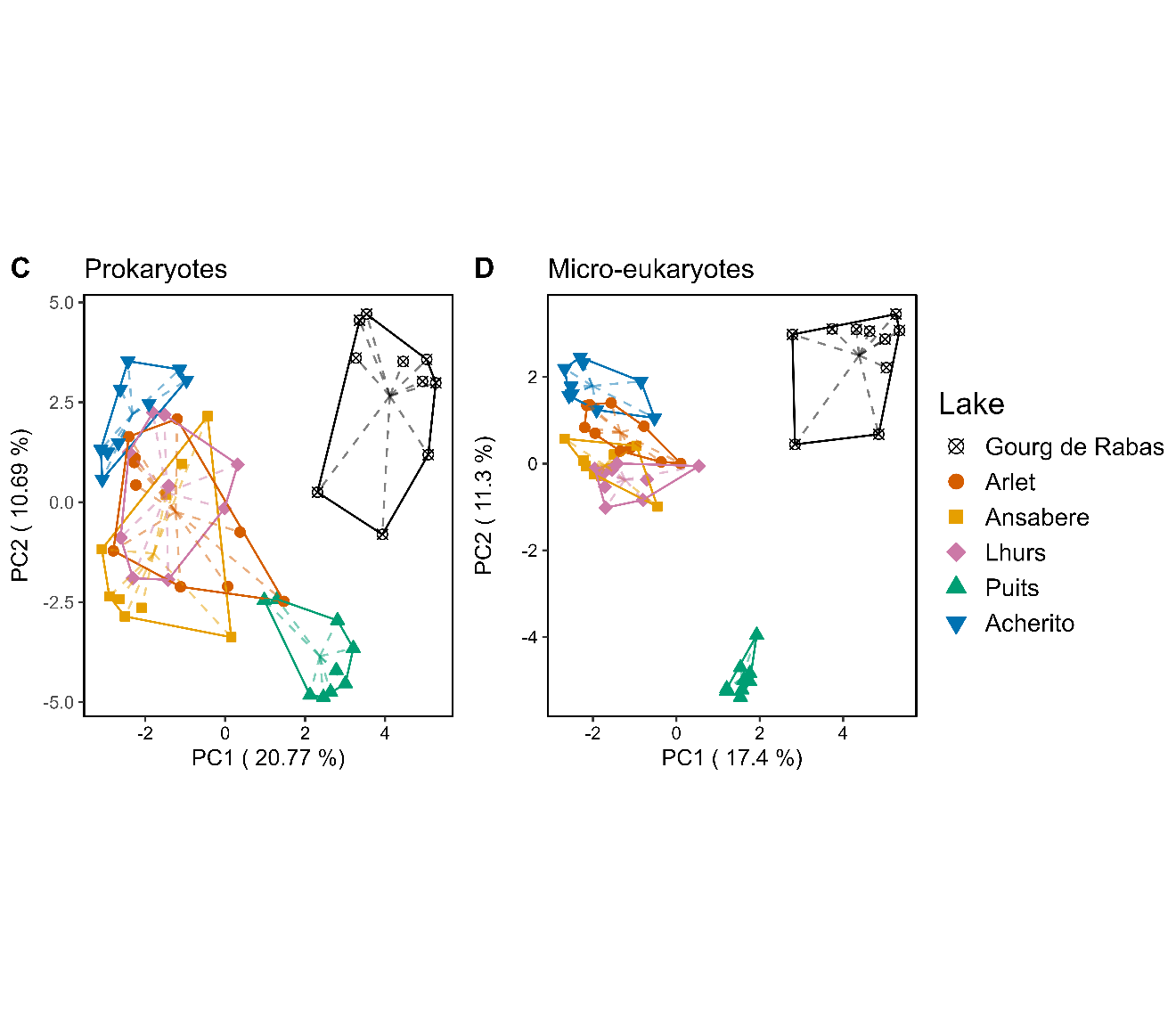


**Figure S2**: **Composition of prokaryotic and micro-eukaryotic assemblages of biofilms sampled in this study.** A and B: composition bar plots of prokaryotic and micro-eukaryotic biofilm assemblages respectively. Samples are facetted by ‘Lake’ and sorted in chronological order. C and D: two-dimensional PCA ordination of clr-transformed data (i.e. PCoA based on Aitchison distance) of prokaryotic and micro-eukaryotic biofilm assemblages

## Figure S3


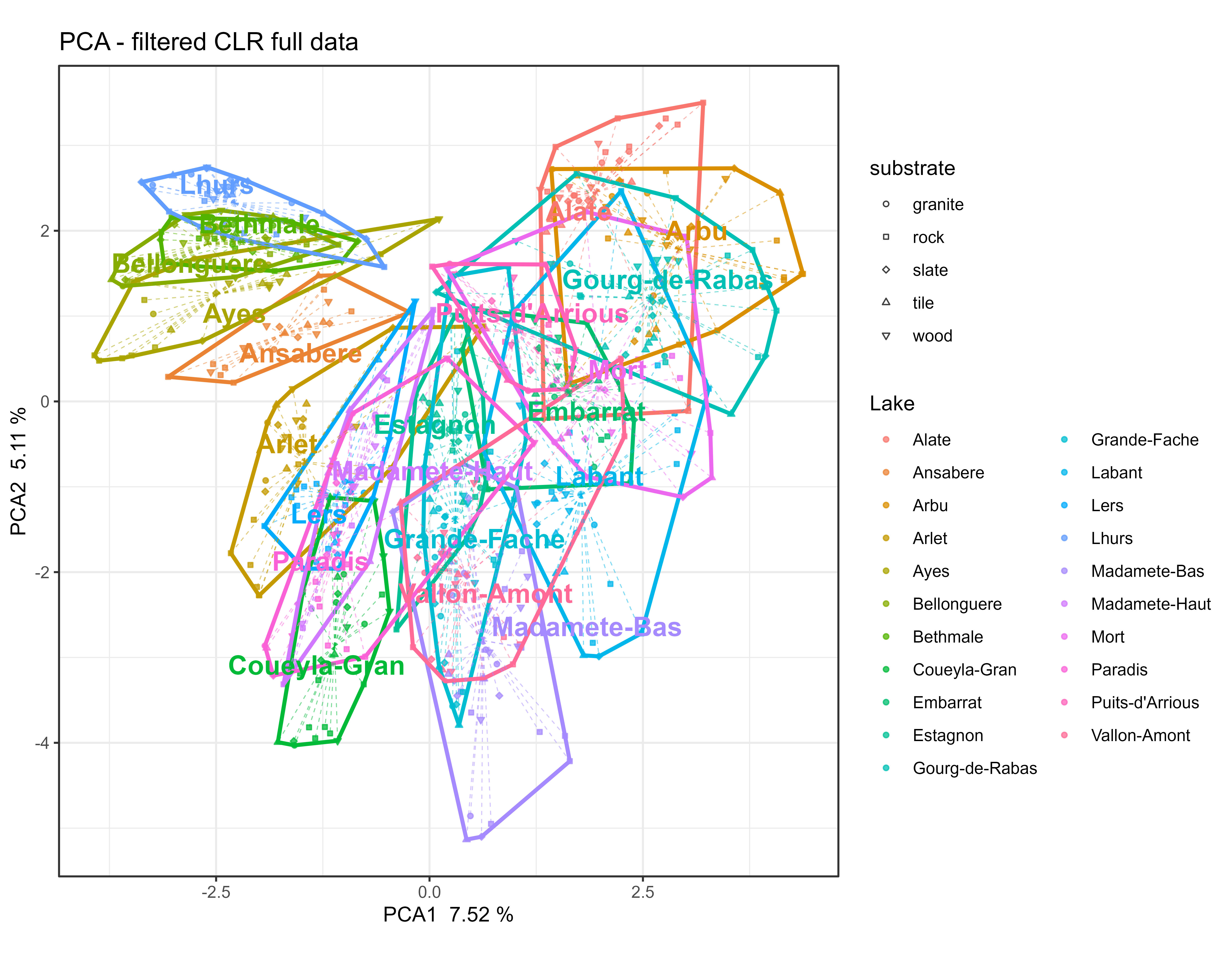


**Figure S3**: **PCA plot of biofilm prokaryotic assemblages (clr transformed data) from 21 different Pyrenean lakes. As part of another study, we placed “artificial substrates” in 21 Pyrenean lakes including Gourg de Rabas.** We used a granite rock, a slate rock, a clay tile rock (different from the ceramic tile used in our experiment) – all mineral substrates –, and a piece of oak wood – an organic substrate. We left them immersed for a year, and then sampled the biofilms and performed 16S rRNA amplicon sequencing. Points represents different biofilm samples, the shape represent the substrate on which the biofilm grew, colored by lakes. A hull has been drawn for each lake to show within-lake biofilm compositional dispersion. All samples from a lake are connected to the lake centroid with dashed lines. The plot clearly shows that lake identity is more important than the type of substrates to shape biofilm prokaryotic communities

## Figure S4


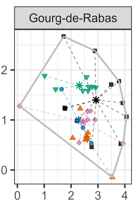

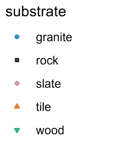


**Figure S4: PCA plot of biofilm prokaryotic assemblages from lake Gourg de Rabas. Points represent different biofilm samples, colored by substrate type.** The natural biofilm communities (“rock”) are not very different from artificial substrates, although a bit more dispersed (likely due to the fact natural rock biofilm samples were collected from 2016 To 2023 whereas biofilms from artificial substrates were collected from 2020 onwards only). Therefore, we assumed that the composition of the biofilms growing on the ceramic tiles that we placed in this lake for our experiment was not very different to natural epilithic biofilms. However, the return to the laboratory (change in abiotic conditions) might have damaged the biofilm.

# Supplementary reference

1. Woodhams DC, Alford RA, Briggs CJ, Johnson M, Rollins-Smith LA. Life-history trade-offs influence disease in changing climates: strategies of an amphibian pathogen. *Ecology* 2008;**89**:1627–39. https://doi.org/10.1890/06-1842.1.

-------------------------------------

End of Document -------------------------------------
